# Validation of Experimental Cooling Performance of Multi-Stage Thin-Film Thermoelectric Devices via Numerical Simulation

**DOI:** 10.3390/mi16060648

**Published:** 2025-05-29

**Authors:** Yu Ning, Longzhou Li, Ping Wei, Shaoqiu Ke, Wanting Zhu, Xiaolei Nie, Danqi He, Mingrui Liu, Wenyu Zhao

**Affiliations:** 1 State Key Laboratory of Advanced Technology for Materials Synthesis and Processing, Wuhan University of Technology, Wuhan 430070, China; yuning@whut.edu.cn (Y.N.); longzhouli@whut.edu.cn (L.L.); zwanting@whut.edu.cn (W.Z.); xiaoleinie@whut.edu.cn (X.N.); hedanqi@whut.edu.cn (D.H.); 2 Hubei Key Laboratory of Photoelectric Materials and Devices, School of Materials Science and Engineering, Hubei Normal University, Huangshi 435002, China; shaoqiuke@whut.edu.cn; 3 Foshan Xianhu Laboratory of the Advanced Energy Science and Technology Guangdong Laboratory, Foshan 528000, China; liumingrui@xhlab.cn

**Keywords:** thermoelectric coolers, thin-film device, finite element simulation, cooling temperature difference

## Abstract

In-plane thermoelectric thin-film cooling devices are considered a promising solution for thermal management in electronic systems. However, the actual cooling performance is far below that of regular bulk cooling devices, making the design of thin-film devices much more difficult. In this work, a numerical analysis of the cooling performance of single-leg thin-film devices and multi-stage cascaded thin-film devices was conducted to understand the depressed cooling performance. The effects of input current, operating environment, substrate, and contact resistance on cooling performance were investigated and compared with the experimental data. The results show that under ideal conditions, including vacuum environment, absence of substrate, and no contact resistance, the maximum cooling temperature difference simulated by the finite element method (105.4 K) closely matches the theoretical value estimated from the *ZT*-based calculation (96.6 K). Under practical conditions, such as within atmosphere and with substrate and contact resistance, the simulated maximum temperature difference (2.1 K) fits well with the experimental value (1.1 K). These findings demonstrate that substrate effects, contact resistance, and operating environment can significantly impair the cooling performance of in-plane film thermoelectric devices, although high-performance thermoelectric materials were used. This study provides a guidance for the design and parameter optimization of thermoelectric thin-film cooling modules.

## 1. Introduction

With the rapid development of integrated circuit technology, the trend toward miniaturization of electronic devices has been significantly accelerated [1,2]. However, miniaturized electronic devices often exhibit extremely high power density and heat flux during operation, which require effective techniques than can dissipate the large amount of heat to the surrounding environment [3,4,5]. Therefore, effective thermal management technologies are crucial for ensuring the stable operation of electronic components. Currently, commonly used thermal management solutions for electronic devices include thermal interface materials [6,7], heat pipe technology [8,9], liquid cooling systems [10,11], and thermoelectric (TE) cooling [12,13]. Among these, TE cooling, an all-solid-state technology with no moving parts, has emerged as a promising solution due to its fast response time, long operational lifetime, high cooling power density, and the potential for miniaturization and thin-film integration [14,15,16,17].

TE devices are fundamentally based on the Peltier effect and use electrons as refrigerant media [18,19]. When a direct current is applied to a TE device, refrigeration appears instantly, with a temperature gradient built at the two ends of the device [20]. The performance of TE cooling mainly depends on the dimensionless figure of merit (*ZT*) of the TE material, defined by *ZT = α*^2^*σT*/*κ*, where *α*, *σ*, *κ*, and *T* are the Seebeck coefficient, electrical conductivity, thermal conductivity, and absolute temperature, respectively [21,22,23]. A large *ZT* of the TE material means a high coefficient of performance for the device [24,25]. In general, a simple equation ${∆T}_{max}={ZT}^{2}/2$ is used to evaluate the maximum cooling temperature difference of a thermoelectric device [26]. However, for a real device, the presence of contact resistance during fabrication and thermal disturbances from the operating environment are unavoidable, and large deviations between experimentally measured maximum cooling temperature difference and theoretical predictions often occur [27].

Researchers have employed finite element analysis to evaluate the cooling performance of TE devices under various parameter conditions, thereby providing a theoretical guidance for the fabrication of more efficient devices. Luo et al. demonstrated through numerical simulations that a reduced thermoelectric leg height and an increased cross-sectional area can significantly improve the cooling performance of TEC devices. However, their study also revealed a considerable deviation between simulated results and experimental measurements [28]. Other factors such as contact resistance, operating environment, thermal conductivity, substrate thickness, and heat source dimensions can also bring about a substantial influence on device performance [29,30,31,32]. To improve the reliability of simulation models, it is necessary to incorporate these practical conditions into consideration during modeling processes, particularly the contact resistance and environmental effects [33,34,35]. However, most work has focused mainly on diverse device numerical models and theoretical calculations used for the optimization and design of TE devices [36,37,38,39,40,41], and systematic validation of these models with experimental data remains scarce.

In this work, the cooling performance of a multiple-stage thin-film TE device was systematically investigated using both theoretical calculations and numerical simulations. The model constructed maintains consistency with the as-assembled device in terms of structural configuration and geometric dimensions, while the material property parameters originate from experimentally measured data [42]. The multi-physics model incorporates Peltier effects, joule heating, and thermal conduction. The effects of input current, material properties, operating environment, supported substrate, and contact resistance on the cooling performance of both single-leg and multi-stage thin-film TE devices are comprehensively analyzed. To validate the reliability of device design, the theoretical prediction, simulation results, and experimental measurements are compared and cross-verified.

## 2. Methods

### 2.1. Device Theoretical Calculation

A complete TE device usually contains multiple pairs of *p*-type and *n*-type legs and metallic electrodes. All the legs are electrically connected by electrodes in series. When an electric current flows through the device, the heat transfer Q from the heat source to the heat sink is primarily facilitated by the *p*-type and *n*-type legs, with each leg type contributing through the Peltier effect, as described by Equation (1) [43]:

(1) $$Q_{p}=\alpha_{p}IT-k_{p}A_{p}\;dT/dx\;Q_{n}={-\alpha }_{n}IT-k_{n}A_{n}\;dT/dx$$

where α, I, k, dT/dx, and T are the absolute Seebeck coefficient, electric current, thermal conductivity, temperature gradient, and absolute temperature, respectively. The legs are of length $L_{p}$ and $L_{n}$ and of cross-section area $A_{p}$ and $A_{n}$ for *p*-type and *n*-type legs, respectively. The Peltier heat is $\alpha_{p}IT$ and $\alpha_{n}IT$ while the heat conduction is $k_{p}A_{p}\;dT/dx$ and $k_{n}A_{n}\;dT/dx$. The rate of heat generation per unit length within each leg due to the Joule effect is $I^{2}/\sigma_{p}A_{p}$ and $I^{2}/\sigma_{n}A_{n}$:

(2) $$-k_{p}A_{p}\cdot d^{2}T/{dx}^{2}=I^{2}/\sigma_{p}A_{p}\;-k_{n}A_{n}\cdot d^{2}T/{dx}^{2}=I^{2}/\sigma_{n}A_{n}$$

where $\sigma_{p}$ and $\sigma_{n}$ are the electrical conductivities.

If setting the boundary condition as ${T=T}_{L}$ at x=0 (i.e., at the heat source) and ${T=T}_{H}$ at $x=L_{p}\;or\;L_{n}$ (i.e., at the heat sink), Equation (2) can be solved by:

(3) $$k_{p}A_{p}\cdot dT/dx=-I^{2}\left(x-\frac{L_{p}}{2}\right)/\sigma_{p}A_{p}+\frac{k_{p}A_{p}(T_{H}-T_{L})}{L_{p}}\;k_{n}A_{n}\cdot dT/dx=-I^{2}\left(x-\frac{L_{n}}{2}\right)/\sigma_{n}A_{n}+\frac{k_{n}A_{n}(T_{H}-T_{L})}{L_{n}}$$

Equations (1) and (3) can be combined to obtain the rate of heat flow at *x* = 0.

(4) $$Q_{p}=\alpha_{p}IT_{L}-k_{p}A_{p}\left(T_{H}-T_{L}\right)/L_{p}-I^{2}L_{p}/2\sigma_{p}A_{p}\;Q_{n}={-\alpha }_{n}IT_{L}-k_{n}A_{n}(T_{H}-T_{L})/L_{n}-I^{2}L_{n}/2\sigma_{n}A_{n}$$

Therefore, the cooling power $Q_{C}$ at the heat source is expressed as:

(5) $$Q_{C}=\left(\alpha_{p}-\alpha_{n}\right)IT_{L}-K\left(T_{H}-T_{L}\right)-I^{2}R/2$$

where the thermal conductance K of the two legs in parallel is:

(6) $$K=k_{p}A_{p}/L_{p}+k_{n}A_{n}/L_{n}$$

and the electrical resistance R of the two legs in series is:

(7) $$R=L_{p}{/\sigma_{p}A}_{p}+L_{n}{/\sigma_{n}A}_{n}$$

The cooling power of the TE device reaches the maximum value if a particular current $I_{p}$ is applied to the device. Such a current can be found when $dQ_{C}/dI=0$:

(8) $$I_{p}=\left(\alpha_{p}-\alpha_{n}\right)T_{L}/R$$

and the maximum cooling power is:

(9) $${\left(Q_{C}\right)}_{max}={\left(\alpha_{p}-\alpha_{n}\right)}^{2}{T_{L}}^{2}/2R-K\left(T_{H}-T_{L}\right)$$

Equation (9) implies that a positive cooling effect cannot be achieved if the temperature difference is too large. In fact, a maximum temperature difference ${∆T}_{max}$ of the π-type TE device can be derived when ${\left(Q_{C}\right)}_{max}=0$.

(10) $${∆T}_{max}={\left(\alpha_{p}-\alpha_{n}\right)}^{2}{T_{L}}^{2}/2KR$$

The figure-of-merit Z of the TE device is defined as:

(11) $$Z={\left(\alpha_{p}-\alpha_{n}\right)}^{2}/KR$$

Hence, Equation (10) can be rewritten as:

(12) $${∆T}_{max}=Z{T_{L}}^{2}/2$$

In this work, three interfaces (heat transfer in solids, electric currents, and TE effect) provided in the COMSOL Multiphysics 6.0 were used. The steady-state energy conservation equation and steady-state electrical current conservation equation were:

(13) $$\rho C_{p}\mu \nabla T+\nabla \left(\alpha T\frac{dI}{dA}-k\nabla T\right)=h\left(T_{E}-T\right)+\frac{d^{2}I}{\sigma dA^{2}}$$

(14) $$\nabla \cdot \left(\frac{dI}{dA}-\sigma \cdot \alpha \cdot \nabla T\right)=0$$

where ρ, $C_{p}$, μ, α, I, A, T, h, $T_{E}$, σ, k, and ∇T are the density, constant-pressure heat capacity, velocity field, Seebeck coefficient, electric current, cross-section area, absolute temperature, atmospheric heat transfer coefficient, ambient temperature, electrical conductivity, thermal conductivity, and temperature gradient, respectively.

### 2.2. Device Simulation and Fabrication

To simplify the model, the following configurations were set for the numerical simulation: (1) all surfaces are adiabatic except for the cold-side and hot-side surfaces; (2) heat losses due to convection and radiation on all surfaces are ignored; (3) contact resistance and contact thermal resistance are ignored; (4) the second type of boundary condition is set on the cold-side surface of the devices; (5) and the third type of boundary condition is set on the hot-side surface of the devices and the external temperature is set to the standard temperature of 293.15 K.

Experimental preparation was as follows. Firstly, mFe/BST/epoxy resin printable paste was prepared through proportion and mixing. Then, MFE/BST/epoxy resin flexible film and BST/epoxy resin flexible film were prepared through screen printing and hot-press curing processes. Then, single-leg thermoelectric cooling devices and cascade thermoelectric cooling thin-film devices were fabricated by laser etching and vacuum evaporation techniques, respectively. These steps ensured the repeatability of the experiment and the reliability of the results. The details can be found in reference [42].

## 3. Results and Discussion

### 3.1. Thermodynamic Behavior of In-Plane TE Devices

**Figure 1:**
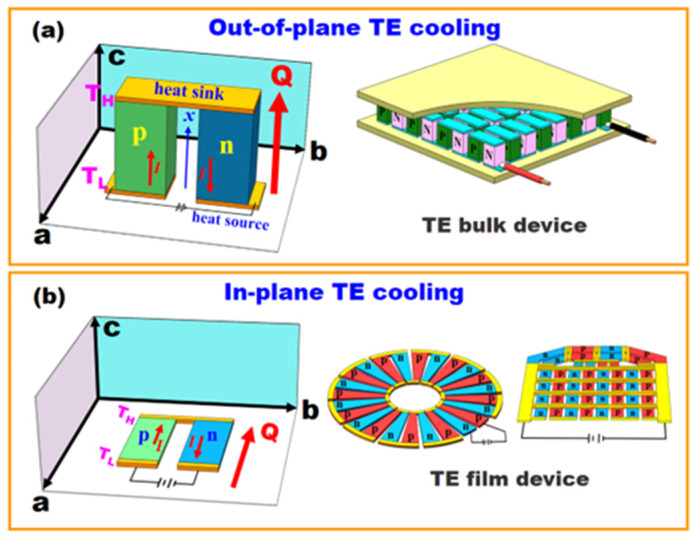
Two types of working modes of TE cooling technology. (**a**) Out-of-plane TE colling, (**b**) In-plane TE cooling.

TE cooling can function in two kinds of working modes: out-of-plane TE cooling (bulk device) and in-plane TE cooling (film device), as shown in Figure 1. The notable feature of out-of-plane TE cooling is that the heat flow (*Q*) is perpendicular to the *ab* plane, while the *Q* is parallel to the *ab* plane for the in-plane TE cooling. As opposed to TE bulk devices, at present, there is no commercial TE film device and no standards for designing, manufacturing, or evaluating TE film devices. Insurmountable factors such as the substrate, contact resistance, and heat exchange with the surrounding environment, which have less influence on TE bulk devices, can have significant impacts on the cooling performance of TE film devices. It has been established that ${∆T}_{max}$ obtained by out-of-plane TE devices is usually much greater than that obtained by in-plane TE devices.

It is apparent that the figure-of-merit Z, as defined by Equation (12), is a characteristic of TE devices, rather than the TE materials, because Z depends on many terms, such as the dimensions of *p*-type and *n*-type legs (length $L_{p}$ and $L_{n}$ and cross-section area $A_{p}$ and $A_{n}$), and the electrical and thermal properties ($\sigma_{p}$, $\alpha_{p}$, $k_{p}$) and ($\sigma_{n}$, $\alpha_{n}$, $k_{n}$) of *n*-type and *p*-type TE materials. For a TE device, the highest value of Z can be reached when the product KR is minimized. Unfortunately, the R rises and the K falls as the ratio of length to cross-section area increases. The importance, however, is to maintain a preferred relationship between L/A in one leg and the other leg. The KR is minimized when:

(15) $$\frac{L_{n}A_{p}}{L_{p}A_{n}}={\left(\frac{\sigma_{n}k_{n}}{\sigma_{p}k_{p}}\right)}^{1/2}$$

Equation (11) is then given as:

(16) $$Z={\left(\alpha_{p}-\alpha_{n}\right)}^{2}/{\left[{\left(k_{n}/\sigma_{n}\right)}^{1/2}+{\left(k_{p}/\sigma_{p}\right)}^{1/2}\right]}^{2}$$

In fact, all the dimension items in Equation (11) can be eliminated mathematically method when the dimensions of *p*-type and *n*-type legs are completely the same, namely $L_{p}=L_{n}$ and $A_{p}=A_{n}$. Equation (11) can be rewritten as:

(17) $$Z=\sigma_{n}\sigma_{p}{\left(\alpha_{p}-\alpha_{n}\right)}^{2}/\left[\left(k_{n}+k_{p}\right)\cdot \left(\sigma_{n}+\sigma_{p}\right)\right]$$

Equations (15) and (16) are rather cumbersome when attempting to find a good TE material candidate, either *p*-type or *n*-type, because this involves the TE properties of both legs. Therefore, the figure-of-merit $z_{n}$ or $z_{p}$ of a single TE material is used, which is defined as:

(18) $$z_{n}=\frac{\sigma_{n}{\alpha_{n}}^{2}}{k_{n}}\;or\;z_{p}=\frac{\sigma_{p}{\alpha_{p}}^{2}}{k_{p}}$$

Only where the *p*-type and *n*-type TE materials are exactly equivalent to each other apart from the sign of the Seebeck coefficient can the figure-of-merit $z_{n}$ or $z_{p}$ be precisely related to Z. In other words, when $\alpha_{p}=-\alpha_{n}$ and $k_{n}/\sigma_{n}=k_{p}/\sigma_{p}$, one can obtain $Z=z_{n}=z_{p}$. Experimentally, it is almost impossible for such a case to occur. In addition, this can also lead to substantial errors by equating the Z of the π-type TE device with the average values of $z_{n}$ and $z_{p}$ from the two legs.

### 3.2. Cooling Performance of TE Devices with Different Structural Designs

**Figure 2:**
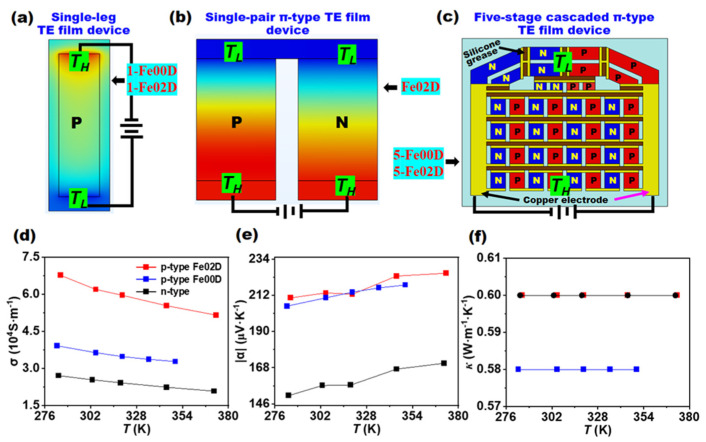
Structure model for (**a**) single-leg TE film device, (**b**) single-pair TE film device, and (**c**) five-stage cascaded TE film device. (**d**–**f**) Measured TE parameters, including (**d**) electrical conductivity, (**e**) absolute Seebeck coefficient, and (**f**) thermal conductivity for *p*-type Bi_0.5_Sb_1.5_Te_3_/epoxy flexible TE film (Fe00D), *p*-type Fe/Bi_0.5_Sb_1.5_Te_3_/epoxy flexible TE film (Fe02D), and *n*-type Bi_2_Te_2.7_Se_0.3_/epoxy flexible TE film used for finite element calculations.

Three device models were established to carry out the study on the cooling performance, as shown in Figure 2: two single-leg TE film devices, a single-pair π-type TE film device, and two five-stage cascaded π-type TE film devices. Five-stage cascaded π-type TE film devices were fabricated in our previous study [37].

The single-leg TE film devices with 4.0 × 20 × 0.06 mm^3^ in size were labeled 1-Fe00D and 1-Fe02D and composed solely of *p*-type Bi_0.5_Sb_1.5_Te_3_/epoxy flexible film (Fe00) and *p*-type Fe/Bi_0.5_Sb_1.5_Te_3_/epoxy flexible film (Fe02), respectively. Here the Fe nanoparticles have a mass percentage of 0.2% in Fe02 film. A single-pair π-type TE film device with 5.37 × 8.3 × 0.15 mm^3^ in size was labeled Fe02D, which was composed of Fe02 film as *p*-leg and Bi_2_Te_2.7_Se_0.3_/epoxy flexible film as *n*-leg. Two five-stage cascaded TE film devices with 45.5 × 69 × 0.15 mm^3^ in size were designed with Fe00 and Fe02 films as *p*-leg and Bi_2_Te_2.7_Se_0.3_/epoxy flexible film as *n*-leg, which had π-type geometric structure and are referred to as 5-Fe00D and 5-Fe02D, respectively. The dimension parameters and the connection pattern of *p*-type and *n*-type legs in two five-stage cascaded TE film devices were determined by the application needs of in-plane heat dissipation of chips in mobile electronics, which has very limited space in the thickness direction.

**Figure 3:**
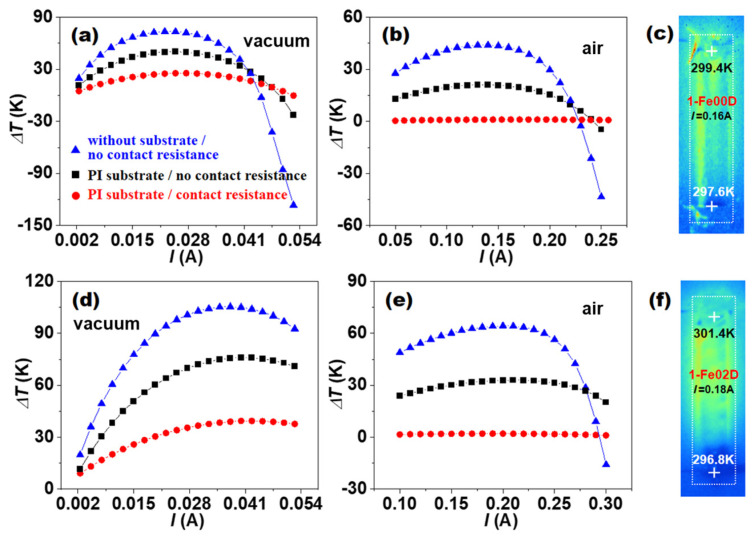
The cooling temperature difference Δ*T* dependence of the current *I* for single-leg TE devices 1-Fe00D and 1-Fe02D under different conditions. (**a**,**b**) 1-Fe00D in vacuum and in air and (**c**) the measured IR thermal image. (**d**,**e**) 1-Fe02D in vacuum and in air and (**f**) the measured IR thermal image. Here *T_H_* = 300 K.

Theoretical cooling performance of 1-Fe00D and 1-Fe02D was simulated by finite element method. Both hot-end and cold-end temperatures *T_H_* and *T_L_* of 1-Fe00D and 1-Fe02D were set at 300 K initially. Maintaining *T_H_* at 300 K with a heat sink, the *T_L_* was calculated under the different operating currents (*I*), and the cooling temperature difference (Δ*T*) was further calculated by Δ*T* = *T_H_* − *T_L_*. The TE properties of *p*-type Fe00, Fe02, and *n*-type films shown in Figure 3d–f have been reported elsewhere [42]. It can be seen that the introduction of Fe nanoparticles can greatly improve the TE performance of Fe00 film. The *n*-type film possessed relatively low TE performance, such as low electrical conductivity and absolute Seebeck coefficient. All these materials had similar thermal conductivity. These measured material parameters were used in the following numerical simulations.

Figure 3 shows the Δ*T* dependence of the *I* for 1-Fe00D and 1-Fe02D. It can be seen that all the Δ*T* of 1-Fe00D and 1-Fe02D first increased and then decreased as the *I* gradually increased. The increase in Δ*T* is due to the Peltier heat (αIT) more than the joule heat ($I^{2}/\sigma A$) under the small-*I* condition, while the decrease in Δ*T* originates from the Peltier heat less than the joule heat under the high-*I* condition. The occurrence of negative Δ*T* originates from the much larger joule heat under the large-*I* condition. The heat absorption induced by the Peltier effect and the heat release induced by the joule effect achieve a dynamic balance under an optimal *I*. The ${∆T}_{max}$ values of 1-Fe00D and 1-Fe02D with no substrate and no contact resistance reach 73.6 K and 105.4 K in vacuum and 44.0 K and 64.4 K in air, respectively. Herein, the 73.6 K and 105.4 K in vacuum are very close to the ${∆T}_{max}$ of 70.3 K for 1-Fe00D and 96.6 K for 1-Fe02D calculated with Equation (12), implying that the simulations are reliable. When the polyimide (PI) substrate 125 μm in thickness attached to the contact resistances is not considered, its ${∆T}_{max}$ is remarkably reduced to 50.3 K and 76.0 K in vacuum and 21.0 K and 33.0 K in air. If the contact resistances of 1-Fe00D and 1-Fe02D are too high to be ignored, the ${∆T}_{max}$ of 1-Fe00D and 1-Fe02D with PI substrate are further reduced to only 25.5 K and 39.4 K in vacuum and 1.1 K and 2.1 K in air, respectively. The theoretical ${∆T}_{max}$ for 1-Fe00D and 1-Fe02D in air are slightly higher than the experimental data of 0.4 K and 1.1 K, as shown in Figure 3c,f [42], which may originate from the lack of consideration of the influence of epoxy resin in the simulations.

These simulation results suggest that the presence of PI substrate, contact resistance, and air environment can greatly affect the cooling performance of single-leg TE film devices, and their negative impact can even exceed the contribution of magnetic nanoparticles in improving the TE cooling performance of films. Therefore, a serious deviation could emerge when simply estimating the ${∆T}_{max}$ value of single-leg TE film devices by Equation (12) if the important influence of the PI substrate, contact resistance, and operating environment were completely ignored. In addition, the size of the heat source and the heat conduction path will also have a certain impact: when the size of the heat source is large, the heat it generates diffuses over a wider range within the simulation area, resulting in a more obvious increase in the medium temperature within a certain distance around the model. When the heat conduction path is short and the thermal conductivity of the heat conduction material is high, heat can be transferred away from the heat source quickly, thereby reducing the temperature accumulation at the heat source [44]. These results also indicate that the manufacturing of TE film devices will face huge challenges, with difficulty far beyond that of traditional TE bulk devices.

According to Goldsmid’s scenario [43], we cannot simply replace the Z in ${∆T}_{max}=Z{T_{L}}^{2}/2$ with the $z_{n}$ or $z_{p}$ of TE materials to calculate the ${∆T}_{max}$ of TE devices. To illustrate how to calculate the ${∆T}_{max}$ values of π-type TE devices, a single-pair π-type TE film device (Fe02D) was modeled with $L_{p}{/A}_{p}=L_{n}{/A}_{n}$. We calculated the Z of Fe02D by Equation (17) and the measured data ($\sigma_{p}$, $\alpha_{p}$, $k_{p}$) and ($\sigma_{n}$, $\alpha_{n}$, $k_{n}$) at 300 K. The ${∆T}_{max}$ of Fe02D in vacuum can be calculated by the equation ${2∆T}_{max}=Z{\left(300-{∆T}_{max}\right)}^{2}$ when $T_{H}=300\;K$. As a result, a ${∆T}_{max}$ value of 60.4 K for Fe02D was obtained. The cooling performance of Fe02D was also simulated by the finite element method and the simulated ${∆T}_{max}$ was 60 K, which fits well with the calculated value.

**Figure 4:**
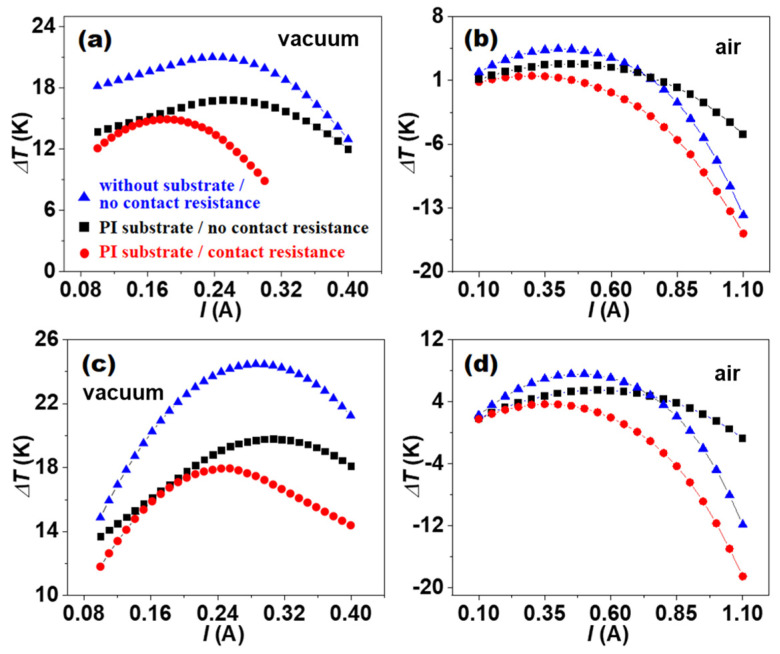
The cooling temperature difference Δ*T* dependence of the current *I* for five-stage cascaded TE devices 5-Fe00D and 5-Fe02D under different conditions. (**a**,**b**) 5-Fe00D in vacuum and in air. (**c**,**d**) 5-Fe02D in vacuum and in air. Here *T_H_* = 300 K.

The ${∆T}_{max}$ values of five-stage cascaded 5-Fe00D and 5-Fe02D were directly simulated since a simple evaluation of ${∆T}_{max}$ by Equation (17) was no longer suitable for the multi-stage cascaded structure. Figure 4 shows the Δ*T* dependence of *I* for 5-Fe00D and 5-Fe02D. It can be seen that all the Δ*T* of 5-Fe00D and 5-Fe02D first increased and then decreased as the *I* gradually increased. Under the high-*I* condition, Δ*T* changed into negative, which originates from the large joule heat ($I^{2}/\sigma A$). When an optimal *I* was applied, the maximum cooling Δ*T* values of 5-Fe00D and 5-Fe02D with no substrate and no contact resistance reached 21.0 K and 24.5 K in vacuum and 4.4 K and 7.6 K in air, respectively. Note that the ${∆T}_{max}$ values of five-stage cascaded devices were much lower than those of single-leg devices, which could be attributed to the much larger heat source used in the cascaded devices. When 5-Fe00D and 5-Fe02D were attached to the PI substrate 125 μm in thickness and their contact resistances were not considered, the simulated ${∆T}_{max}$ values were remarkably reduced to 16.8 K and 19.8 K in vacuum and 2.8 K and 5.5 K in air, respectively.

**Figure 5:**
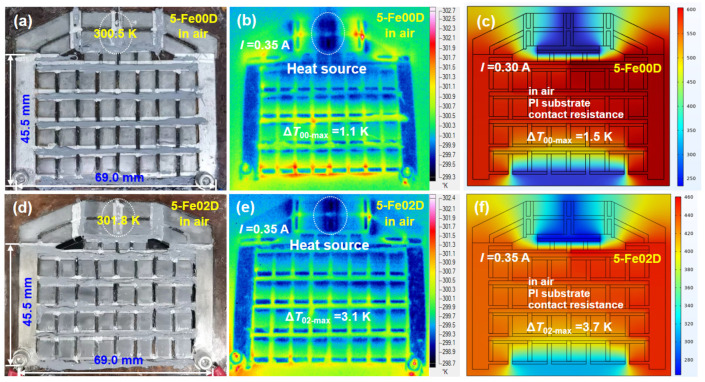
Cooling performance measurement and simulation of five-stage cascaded film devices Fe00D and Fe02D. Real photo (**a**), IR thermal image (**b**), and temperature distribution map (**c**) of 5-Fe00D. Real photo (**d**), IR thermal image (**e**), and temperature distribution map (**f**) of 5-Fe02D. The IR thermal image measurements were performed in air. The simulation conditions included PI substrate, contact resistance, and in air.

If the contact resistances of 5-Fe00D and 5-Fe02D are too high to be ignored, the ${∆T}_{max}$ values of 5-Fe00D and 5-Fe02D with PI substrate are further reduced to only 14.9 K and 18.0 K in vacuum and 1.5 K and 3.7 K in air, respectively. As shown in Figure 5, the two cascaded film devices were much larger in both length and width. The IR thermal images indicated that the measured ${∆T}_{max}$ values for 5-Fe00D and 5-Fe02D were 1.1 K and 3.1 K, respectively, with heat source used to simulate the chip power. The simulated ${∆T}_{max}$ values closely fitted the experimental data [42], suggesting that the small ${∆T}_{max}$ values obtained on real devices were mainly limited by the film device conditions, such as substrate, contact resistance, and circumstance.

These theoretical results of five-stage cascaded π-type 5-Fe00D and 5-Fe02D above clearly demonstrate that besides the $z_{n}$ and $z_{p}$ of *n*-type and *p*-type TE films, the PI substrate, dimension parameters, operating environment, and connection pattern of *p*-type and *n*-type legs have important impact on the ${∆T}_{max}$ through the in-plane heat dissipation by in-plane TE cooling based on TE film devices. The ${∆T}_{max}$ values of five-stage cascaded device5-Fe02D are smaller than those of single-leg devices, which also originates from the specific dimension parameters and connection pattern of *p*-type leg and *n*-type leg in five-stage device. However, the design of multiple stages is required by the application for achieving in-plane heat dissipation function.

It can be seen that the theoretical ${∆T}_{max}$ values of π-type film devices Fe02D, 5-Fe00D, and 5-Fe02D are relatively low, with the advantage of the high *zT* value of 1.4 for the *p*-type Fe/BST/epoxy flexible film not shown in the devices. The reason is that the *zT* values of *n*-type Bi_2_Te_2.7_Se_0.3_/epoxy flexible film as *n*-leg are too low—only about 0.3 at 300 K. How to significantly improve the *zT* of *n*-leg TE film needs more investigation in the future research.

In practical applications, we evaluated the application cases of TE thin-film devices in the heat dissipation of mobile electronic devices. Under specific working conditions, the device can reduce the chip temperature by approximately 3 °C, effectively improving the heat dissipation performance of the equipment. This result indicates that TE thin-film devices have potential in solving the heat dissipation problem of high-power-density electronic equipment.

## 4. Conclusions

The maximum cooling Δ*T* values of single-leg and cascaded TE film devices were evaluated by finite element simulations with consideration of different boundary conditions including the substrate, contact resistance, and operating environment. Under ideal conditions, the maximum cooling temperature difference calculated using the finite element method (105.4 K) closely matches the theoretical value estimated from the *ZT*-based calculation (96.6 K). When considering the atmosphere, substrate, and contact resistance, the simulated maximum temperature differences were also closely aligned with the experimental values, indicating the importance of these boundary conditions in impacting cooling performance, although the addition of magnetic Fe nanoparticles was largely effective in improving cooling performance. This work provides a guideline for the fabrication of high-performance TE film devices, which are still prone to small maximum cooling Δ*T* values.

## Data Availability

The original contributions presented in the study are included in the article, further inquiries can be directed to the corresponding authors.

## Abbreviations

The following abbreviations are used in this manuscript:

TE	thermoelectric
PI	polyimide
